# **Fermentative hydrogen production from glucose and starch using pure strains and artificial co-cultures of***Clostridium spp.*

**DOI:** 10.1186/1754-6834-5-35

**Published:** 2012-05-22

**Authors:** Julien Masset, Magdalena Calusinska, Christopher Hamilton, Serge Hiligsmann, Bernard Joris, Annick Wilmotte, Philippe Thonart

**Affiliations:** 1 Walloon Centre of Industrial Biology, Boulevard du Rectorat 29, Liège, B4000, Belgium; 2 Centre for Protein Engineering, Bacterial physiology and genetics, University of Liège, Allée de la Chimie 3, Liège, B4000, Belgium

**Keywords:** *Clostridium spp*., Fermentative H_2_ production, qPCR

## Abstract

**Background:**

Pure bacterial strains give better yields when producing H_2_ than mixed, natural communities. However the main drawback with the pure cultures is the need to perform the fermentations under sterile conditions. Therefore, H_2_ production using artificial co-cultures, composed of well characterized strains, is one of the directions currently undertaken in the field of biohydrogen research.

**Results:**

Four pure *Clostridium* cultures, including *C. butyricum* CWBI1009, *C. pasteurianum* DSM525, *C. beijerinckii* DSM1820 and *C. felsineum* DSM749, and three different co-cultures composed of (1) *C. pasteurianum* and C. *felsineum*, (2) *C. butyricum* and *C. felsineum*, (3) *C. butyricum* and *C. pasteurianum*, were grown in 20 L batch bioreactors. In the first part of the study a strategy composed of three-culture sequences was developed to determine the optimal pH for H_2_ production (sequence 1); and the H_2_-producing potential of each pure strain and co-culture, during glucose (sequence 2) and starch (sequence 3) fermentations at the optimal pH. The best H_2_ yields were obtained for starch fermentations, and the highest yield of 2.91 mol H_2_/ mol hexose was reported for *C. butyricum*. By contrast, the biogas production rates were higher for glucose fermentations and the highest value of 1.5 L biogas/ h was observed for the co-culture (1). In general co-cultures produced H_2_ at higher rates than the pure *Clostridium* cultures, without negatively affecting the H_2_ yields. Interestingly, all the *Clostridium* strains and co-cultures were shown to utilize lactate (present in a starch-containing medium), and *C. beijerinckii* was able to re-consume formate producing additional H_2_. In the second part of the study the co-culture (3) was used to produce H_2_ during 13 days of glucose fermentation in a sequencing batch reactor (SBR). In addition, the species dynamics, as monitored by qPCR (quantitative real-time PCR), showed a stable coexistence of *C. pasteurianum* and *C. butyricum* during this fermentation.

**Conclusions:**

The four pure *Clostridium* strains and the artificial co-cultures tested in this study were shown to efficiently produce H_2_ using glucose and starch as carbon sources. The artificial co-cultures produced H_2_ at higher rates than the pure strains, while the H_2_ yields were only slightly affected.

## Background

Hydrogen is regarded as a future energy vector for transportation and stationary power. However, its current production is heavily dependent on fossil fuels, yielding globally 40% of H_2_ from steam reforming of methane and another 48% from crude oil and coal. By contrast, biohydrogen production offers a sustainable alternative and by utilizing renewable carbon resources can be considered as a CO_2_ offset [1].

Hydrogen can be produced biologically by four different processes: direct and indirect biophotolysis, photofermentation and dark fermentation. Fermentative hydrogen production not only provides higher gas production rates compared to photosynthetic processes, but is also light independent and can utilize various carbon sources, including wastewaters [2].

Many agricultural and food-industry wastes and wastewaters contain large quantities of carbohydrates and proteins. However, their complex nature can adversely affect their digestibility for the fermentative organism(s) present in a H_2_ producing bioreactor [3]. Naturally occurring microbial consortia derived from different sources, such as compost, anaerobic sludge or soil, are frequently used for H_2_ production. On one hand due to the metabolic flexibility, provided by the different members of the community, they are vital to the waste treatment systems, as they can tolerate different substrates and environmental conditions [4]. On the other hand the same metabolic flexibility can undermine the H_2_ production efficiency by initiating and sustaining other metabolic pathways, *e.g.* increased lactate formation by lactic acid bacteria [5]. Additionally it has been shown that within a complex and poorly defined population, bacterial successions can occur even within a batch culture, and that this can significantly impact H_2_ production [6,7].

In comparison to the use of natural, mixed bacterial populations, pure strain cultures were shown to produce H_2_ at higher yields [8]. However, the main drawback of the use of pure cultures is the necessity to maintain the fermentations under sterile conditions. In fact the autochthonous bacteria present in the waste materials can easily overgrow the pure strain used to produce H_2_ in a bioreactor. Therefore, the use of artificial microbial co-cultures and consortia, composed of pre-defined and well characterized species, has attracted particular interest in the developing biohydrogen industry. Co-cultures can perform complex functions, such as simultaneous pentose or hexose consumption, which generally cannot be performed by a single species [9]. Being potentially more robust to changes in environmental conditions, microbial co-cultures can resist periods of nutrient limitation better; what is typically combined with the exchange of metabolites between the different bacteria [10]. Another feature is biomass concentration with the formation of aggregates, which occurs more readily in co-cultures than with a single strain culture [11]. However, until now, the creation of a stable artificial community composed of three or more members has been problematic due to the diverse rates of growth of the different members and the subsequent imbalance between the consumption and production of metabolites [12]. To date, a stable coexistence of community members over a long period of time has only been reported for a few H_2_-producing artificial consortia and co-cultures [13-16].

Therefore this study has two goals. The first is to characterize H_2_ production by four *Clostridium* strains (*C. beijerinckii* DSM 1820, *C. butyricum* CWBI 1009, *C. pasteurianum* DSM 525 and *C. felsineum* DSM 794) grown as pure cultures with different substrates. The second goal is to develop stable H_2_ producing co-cultures composed of two different *Clostridium spp.* A simple three-step approach, consisting of three fermentation sequences, was used to first assess the optimal pH for H_2_ production for pure strains and for co-cultures with glucose (sequence 1). Then the potential of the pure cultures and co-cultures to stably produce H_2_ using different carbon sources was evaluated by performing glucose (sequence 2) and starch fermentations (sequence 3) at a controlled optimal pH. In order to assess the metabolic interactions between the different strains, the metabolites produced were monitored by HPLC. The potential of the co-culture, composed of *C. butyricum* and *C. pasteurianum* (co-culture 3), to grow consistently and produce H_2_ was also evaluated during a 13-day glucose fermentation in a sequencing batch bioreactor (SBR). The dynamics of the two strains was monitored throughout the fermentation using a previously optimized qPCR assay based on quantification of the *recA* and *gyrA* marker genes [17].

## Results and Discussion

In the first part of this study a strategy of three-culture sequences was developed to characterize hydrogen production by four pure *Clostridium* strains (*C. beijerinckii* DSM 1820, *C. butyricum* CWBI 1009, *C. pasteurianum* DSM 525 and *C. felsineum* DSM 794), and three co-cultures composed of two closely related clostridia; *i.e*. co-culture (1) with *C. pasteurianum* and *C. felsineum*, co-culture (2) with *C. butyricum* and *C. felsineum*, and co-culture (3) with *C. pasteurianum* and *C. butyricum*. Two strains, namely *C. butyricum* and *C. beijerinckii,* have been widely investigated for H_2_-production in bioreactors and they were shown to ferment a variety of different carbon sources, including pure sugars (*e.g.* glucose, starch, sucrose, galactose [18-22] palm oil [23] yeast wastes [24] or sugar cane juice [25]. *C. pasteurianum* has been often detected as a dominant strain in sludge-derived H_2_-producing mixed cultures [26]. By contrast the application of *C. felsineum* for H_2_ production has not yet been reported.

The hydrogen production potential of the pure strains and co-cultures was evaluated in the same 20 L SBRs. The first sequence was conducted without pH control using glucose as a carbon source. By monitoring the hydrogen production rate for each pure strain and co-culture, the optimal pH for H_2_ production was determined. The second and the third sequences were carried out at optimal pH values, using either glucose or starch as fermentative substrates.

Additionally in the second part of the study, co-culture (3) with *C. pasteurianum* and *C. butyricum,* was used to produce hydrogen over a longer period (13 days) in a SBR, maintained at fixed pH using glucose as the carbon source.

### Single batch fermentation to evaluate the optimal pH for H_2_ production (Sequence 1)

pH is considered as one of the key factors affecting H_2_ production and metabolic pathways in *Clostridium spp.*[20,21,27]. Therefore during the first sequence with glucose the optimal pH for H_2_ production was determined for the pure strains and for the co-cultures. Previously the optimal pH for H_2_ production was determined by comparing the H_2_ yields obtained in different batch fermentations (multi-stage fermentation approach) performed at different fixed pH values [21,27]. However, this method is time consuming and requires several batch fermentations in order to increase accuracy. In our study the optimal pH of the medium was defined in a single glucose batch fermentation with the initial pH of the medium adjusted to 7.3. Due to the production of acidic metabolites, mainly formate, lactate, acetate and butyrate, the pH of the medium decreased progressively reaching 4.8 ±0.2 at the end of the fermentation. The decreasing pH of the medium triggered H_2_ production. The optimal pH was defined as the pH value at which the biogas production rate reached a maximum (Table 1). This approach also made it possible to determine the lower inhibiting pH value at which hydrogen production ceased completely.

**Table 1:** **Optimal pH ranges for H**_**2**_**production by pure** ***Clostridium*** **strains and co-cultures determined during one-stage glucose fermentation (first sequence) at unregulated pH**

**Strain/ co-culture**	**pH value**	
	**Optimal**	**Inhibitory**
*C. beijerinckii* DSM 1820	6.7	5.2
*C. butyricum* CWBI 1009	5.15	4.7
*C. pasteurianum* DSM 525	5.4	4.6
*C. felsineum* DSM 794	5.5	5
Co-culture 1	5.3	4.7
Co-culture 2	5.3	4.6
Co-culture 3	5.3	5.0

The optimal pH values defined for *C. butyricumC. felsineum* and *C. pasteurianum* were similar, on average 5.35 ±0.15. This pH value is similar to the optimal pH of 5.5 observed for the best H_2_ production by *C. butyricum* EB6 [28]. By contrast the optimal pH for *C. beijerinckii* was pH 6.7 (Table 1). Furthermore, its H_2_ production was inhibited at pH 5.2, which overlaps with the optimal pH values for the three other *Clostridium* strains selected. Therefore, due to its very different pH requirements, *C. beijerinckii* was not used in any of the three two-strain co-cultures. Additionally, this species has been shown to inhibit the growth of other bacteria, including the *Clostridium* species analysed in this study (data not shown), probably by producing bacteriocin and circularin A [16].

**Figure 1:**
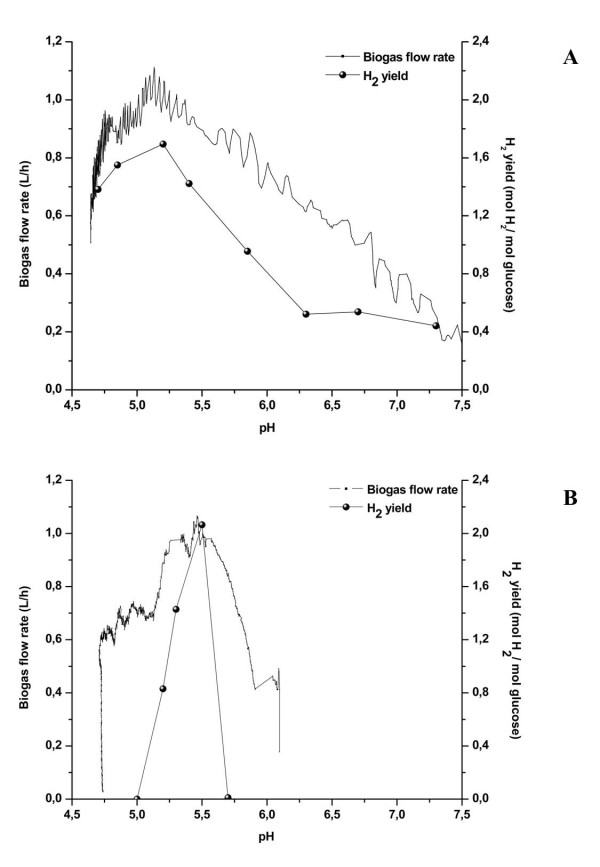
**Comparison of a single-batch fermentation approach (monitoring the biogas flow rate) versus a multi-stage fermentation approach (monitoring the H**_2_**yield obtained at different pH values) to evaluate the optimal pH for H**_2_**production.** (**A**)-*C. butyricum*; (**B**)-*C. pasteurianum*.

In order to validate the data obtained, we performed a comparison between the optimal pH values for H_2_ production determined for *C. butyricum* CWBI 1009 and for *C. pasteurianum* DSM 525 by using a previously described multi-stage fermentation approach (Figure 1A and 1B) [21]. Finally the optimal pH values determined for the two strains with the two different approaches were similar; r indicating that the new single batch fermentation approach provides a fast and accurate means of determining the optimal pH for H_2_ production.

To determine the optimal pH for each co-culture, the same single batch fermentation approach was used. For all three co-cultures the optimal pH for H_2_ production with glucose was 5.3 (Table 1). The inhibiting pH values for co-cultures (1) with *C. pasteurianum* and *C. felsineum* and (2) with *C. butyricum* and *C. felsineum* were found to be 4.7 and 4.6, respectively. However for co-culture (3) with *C. butyricum* and *C. pasteurianum,* the inhibiting pH of 5.0 was very close to the optimal one (5.3). Therefore, use of this co-culture in large bioreactors, in which significant pH variations occur, would require care to avoid inhibition of H_2_ production due to a small shift in pH [29]. A narrow optimal pH range for H_2_ production had already been suggested in previous studies, which reported that even small pH variations caused a shift in the metabolic pathways leading to a reduction in the final H_2_ yield [21,30].

### Metabolites and H_2_ production during glucose batch fermentations with unregulated pH (Sequence 1)

**Figure 2:**
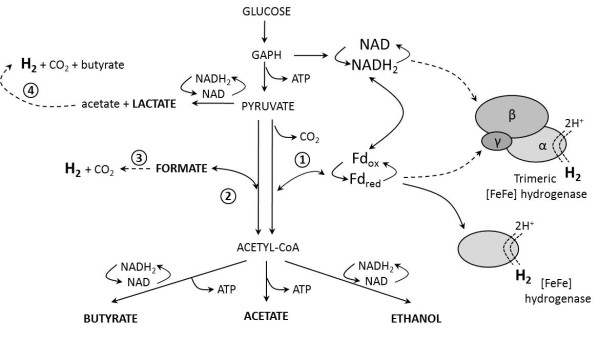
**A simplified overview of the main metabolic pathways involved in H**_**2**_**production by*Clostridium spp.*** Dashed lines indicate hypothetical pathways. 1-PFOR, 2-PFL, 3-[FeFe] TR(M2) hydrogenase [32], 4-pathway proposed by Matsumoto *et al*., [33], Fd_ox/red_-ferredoxin oxidized/reduced.

Clostridia metabolize glucose to pyruvate via the glycolysis pathway (Figure 2). Subsequently, pyruvate is converted to lactate and acetyl-CoA. The latter reaction is driven either by pyruvate:formate lyase (PFL) resulting in formate production, or by pyruvate:ferredoxin oxidoreductase (PFOR) with co-generation of reduced ferredoxin (Fd). Reduced Fd is an electron donor for hydrogenases, which are the H_2_-generating enzymes [31]. Both pathways lead to acetyl-CoA that can be further converted mainly to butyrate, acetate and ethanol. Therefore, it can be assumed that the hydrogen production rate will be affected when high levels of formate are produced, since no reduced Fd is generated.

Comparing the pure strains and the co-cultures, there was a significant variation in the pattern of the fermentation metabolites obtained during glucose batch fermentations with unregulated pH conditions (Table 2). The glucose fermentations resulted primarily in the production of formate for the pure strains of *C. felsineum* (12.14 mM) and formate (18.48 mM ) and butyrate (16.58 mM) for *C. butyricum*. High production of both formate and butyrate was also characteristic for co-culture (2) with *C. butyricum* and *C. felsineum* (13.44 mM and 11.25 mM) and co-culture (3) with *C. butyricum* and *C. pasteurianum* (8.00 mM and 9.12 mM). Acetate molar concentrations were similar for the pure cultures of *C. butyricum* and *C. pasteurianum* (10.01 ±0.81 mM) and for the three co-cultures (8.89 ± 0.47 mM); suggesting that during unregulated pH fermentation acetate production may be a common path for ATP synthesis [34]. By contrast, for pure *C. beijerinckii* and *C. felsineum* strains acetate was the metabolite with the lowest yield (3.38 ± 0.23 mM). For *C. pasteurianum* as well as for co-culture (1), with *C. pasteurianum* and *C. felsineum*, the product composition changed yielding higher amounts of lactate (12.78 mM and 11.81 mM for the pure strain and the co-culture, respectively) and lower amounts of formate (0.57 mM and 3.09 mM for the pure strain and the co-culture, respectively). Surprisingly, co-culture (1) also provided the highest H_2_ yield (Table 2), despite the fact that lactate is the most unfavourable by-product, since it drains away electrons required for H_2_ production [35]. On one hand it was suggested that the activation of lactate production could be an additional catabolic pathway, enabling the bacterium to cope with the excesses of carbon and NADH produced during the GAPDH step (glyceraldehyde 3-phosphate dehydrogenase; Figure 2) of glycolysis [36]. On the other hand it had already been demonstrated that under different conditions, *e.g.* with iron limitation, *C. pasteurianum* fermented glucose to lactate without affecting H_2_ production, mainly at the expense of butyrate [37]. As stated above, *C. pasteurianum* produced lactate as the main metabolite, while its butyrate production was 9% and 54% lower than that for *C. beijerinckii* and *C. butyricum*, respectively (Table 2). Similarly, co-culture (1) with *C. pasteurianum* and *C. felsineum* produced the lowest amount of butyrate (3.90 mM) and its lower formate production and increased H_2_ yield could suggest that pyruvate was converted to acetyl-CoA via the PFOR pathway, rather than via PFL (Figure 2). By contrast, despite the high molar concentrations of acetate and butyrate, commonly associated with higher H_2_ yields [38], the H_2_ yields obtained for culture (2) with *C. butyricum* and *C. felsineum* and culture (3) with *C. butyricum* and *C. pasteurianum* were lower (Table 2); due to the preferential formation of acetyl-CoA by the PFL enzyme, as could be expected given the increased formate production.

**Table 2:** Fermentation end products (molar concentrations) obtained during glucose fermentation at unregulated pH (first sequence)

**Strain/co-culture**	**Formate (mM)**	**Lactate (mM)**	**Acetate (mM)**	**Butyrate (mM)**	**H**_**2**_**yield (mol H**_**2**_**/mol glucose)**	**Biogas production rate (L biogas/ h)**^**a**^
*C. beijerinckii* DSM 1820	3.92	5.28	3.15	8.33	1.45	0.38
*C. butyricum* CWBI 1009	18.48	3.44	9.20	16.58	0.97	0.34
*C. pasteurianum* DSM 525	0.57	12.78	10.82	7.65	0.66	1.03
*C. felsineum* DSM 794	12.14	0.61	3.61	1.80	0.62	0.93
Co-culture 1	3.09	11.81	8.45	3.89	1.61	0.92
Co-culture 2	13.44	1.79	9.55	11.25	1.02	1.02
Co-culture 3	8.0	3.61	8.67	9.12	1.33	1.4

^a^ Maximum biogas production rate.

### Metabolites and H_2_ production during glucose batch fermentations at fixed optimal pH (Sequence 2)

**Figure 3:**
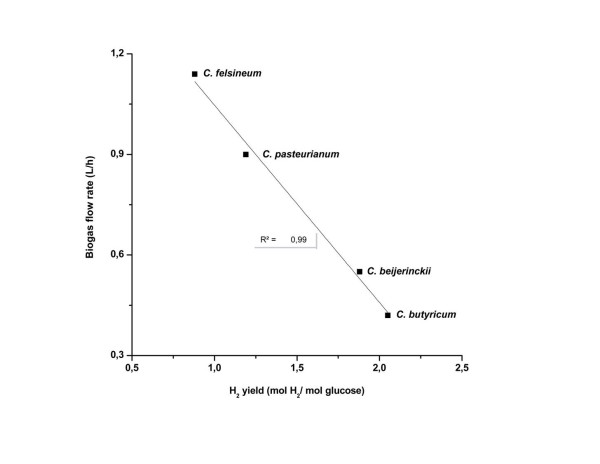
**Inversely proportional relation between H**_**2**_**yield and production rate for pure** ***Clostridium*** **cultures.**

After the first sequence, in which the correct pH was determined for each pure strain and co-culture, 3 L of the medium (*i.e.* 15% of the working volume) were replaced with the same amount of fresh MDT medium containing glucose at a final concentration of 5 g/ L. During this sequence the optimal pH for each pure strain and co-culture was kept constant (Table 1). The *C. butyricum* CWBI 1009 strain provided the highest H_2_ yield of 2.1 mol H_2_/ mol glucose. However, the maximum biogas production rate (0.42 L of biogas /h) was significantly lower than that for the other three pure strain cultures (Table 3). Interestingly, a strong inversely proportional correlation between biogas production rates and H_2_ yields was observed for the pure *Clostridium* strains (Figure 3). The corresponding increase in H_2_ yield with decreasing biogas production rate can be explained by the lower H_2_ partial pressure in the fermentation medium, as previously discussed by other authors [18,39]. Additionally, high H_2_ partial pressure has been shown to have an inhibitory effect on hydrogenases, with a metabolic shift towards the production of other reduced metabolites, *e.g.* lactate, ethanol or formate [40]. Accordingly, *C. felsineum*, which provided the highest biogas production rate and the lowest yield during glucose fermentation at fixed pH, also produced the highest levels of lactate and formate (Table 3).

**Table 3:** Fermentation end products (molar concentrations) obtained during glucose fermentation performed at fixed optimal pH values (second sequence)

**Strain/ co-culture**	**Formate (mM)**	**Lactate (mM)**	**Acetate (mM)**	**Butyrate (mM)**	**H**_**2**_**yield (mol H**_**2**_**/ mol glucose)**	**Biogas production rate (L biogas/ h)**^**a**^
*C. beijerinckii* DSM 1820	1.69	−1.85	4.48	16.99	1.88	0.55
*C. butyricum* CWBI 1009	−0.05	2.74	11.44	17.13	2.10	0.42
*C. pasteurianum* DSM 525	8.66	4.17	11.24	11.39	1.19	0.90
*C. felsineum* DSM 794	4.29	23.42	3.08	4.49	0.88	1.14
Co-culture 1	0.21	4.73	6.20	5.73	1.71	1.50
Co-culture 2	0.74	4.18	8.95	10.76	1.62	1.10
Co-culture 3	0.93	4.57	7.25	8.46	2.12	1.46

^a^ Maximum biogas production rate.

Maintaining the culture at fixed optimal pH gave rise to a 10% increase in the H_2_ yield compared to unregulated pH conditions for co-culture (1) with *C. pasteurianum* and *C. felsineum*, and provided an increase of as much as 60% for co-cultures (2) and (3) (*C. butyricum* &*C. felsineum* and *C. butyricum* &*C. pasteurianum,* respectively*)* (Table 3). The 2.12 mol H_2_/mol glucose yield obtained by co-culture 3 was comparable to the results obtained for *C. butyricum* grown separately, but the co-culture 3 biogas production rate was 1.6 and 3.5 fold higher than that obtained with the pure *C. pasteurianum* and *C. butyricum* strains, respectively (Table 3).

Compared to the previous sequence, the composition of the fermentation end products changed radically for both the pure strains and for the three co-cultures, yielding on average a higher proportion of acetate and butyrate in the total soluble metabolites, when the pH was fixed at the optimal value (Table 3). For *C. beijerinckii*, *C. butyricum* and *C. pasteurianum* the production of lactate decreased significantly, while for *C. felsineum* it increased. The increased lactate production, observed for co-culture (1) with *C. pasteurianum* and *C. felsineum* during the previous batch glucose fermentation with unregulated pH, was no longer present and the three co-cultures produced similar amounts of lactate (4.49 ±0.23 mM) during this fermentation sequence. A decrease in formate production, ranging from 8 to 18 fold, was observed at fixed pH for co-cultures (3) with *C. butyricum* and *C. pasteurianum*, and (2) with *C. butyricum* and *C. felsineum*. Additionally, the increased H_2_ yield for the three co-cultures suggested that pyruvate was mainly metabolized to acetyl-CoA via PFOR (Figure 2).

### Metabolites and H_2_ production during starch batch fermentations at fixed optimal pH (Sequence 3)

Simple sugars, *e.g.* glucose or lactose, are the preferred carbon sources for H_2_ producing fermentative bacteria, due to their fast biodegradability [20,22,41]. However, pure carbohydrates are far too expensive for use as feedstock and renewable waste material is therefore an attractive potential alternative for the sustainable production of hydrogen [3,42]. Consequently, during the third fermentation sequence with removal/addition of 3 L of the MDT medium, the ability of the pure *Clostridium* strains and the co-cultures to grow on different carbon sources was further assessed using starch (5 g/ L), which is present at high levels in many food industry and agricultural wastes.

Pure *C. butyricum* and *C. pasteurianum* cultures consumed respectively 62.8% and 57.4% of the starch initially added to the medium (after 46.5 h ±1). These results seem to contradict the findings of Chen *et al*., where the authors had to employ a starch pre-treatment step in order to accelerate hydrogen production [43]. In their work *C. pasteurianum* (different indigenous isolates from Taiwan) was reported to be unable to use starch, neither for the cell growth nor H_2_ production.

Of the other two pure strains, *C. beijerinckii* consumed merely 13.4% of the starch after 71 h of fermentation, and *C. felsineum* was unable to use starch as a fermentative carbon source. By contrast with these results, another strain of *C. beijerinckii,* namely *C. beijerinckii* AM21B isolated from termites, was reported to efficiently consume starch with concomitant production of H_2_[44].

By contrast with the pure strain cultures, starch was shown to be easily degraded by the three co-cultures and was entirely consumed after 47.5 h ±0.5 by co-culture (1) with *C. pasteurianum* and *C. felsineum* and by co-culture (3) with *C. butyricum* and *C. pasteurianum,* without the need for any pre-treatment such as acid or enzymatic hydrolysis. Co-culture (2) with *C. butyricum* and *C. felsineum* required 70 h of fermentation to consume all the starch, probably due to a lower hydrolysis rate.

For the pure strains the biogas production rates were lower than those obtained during the previous glucose fermentation sequence, probably due to the lower biodegradability of starch and the additional hydrolysis step necessary to release the fermentable sugars. As a result, the resulting H_2_ partial pressure may have been lower, thus favouring higher H_2_ yields, which increased by an average of 44% ±6 for *C. butyricum* and *C. pasteurianum*. Even though *C. beijerinckii* did not consume significant amounts of starch the strain was shown to produce H_2_, using both formate and lactate produced during the previous glucose fermentation sequences (Table 4). Also, even if *C. felsineum* was unable to consume any starch, it nevertheless produced H_2_ during this fermentation sequence, mainly from the lactate present in the medium. The ability of these bacteria to reconsume formate and lactate indicates the existence of new metabolic pathways in clostridia (Figure 2, indicated by dashed line). It opens new perspectives for a better substrate utilization, resulting in increased hydrogen production rates and/or yield [45]_._

**Table 4:** Fermentation end products (molar concentrations) obtained during the starch fermentation performed at fixed optimal pH values (third sequence)

**Strain/co-culture**	**Formate (mM)**	**Lactate (mM)**	**Acetate (mM)**	**Butyrate (mM)**	**H**_**2**_**yield (mol H**_**2**_**/mol hexose)**	**Biogas production rate (L biogas/ h) **^**a**^
*C. beijerinckii* DSM 1820	−4.34	−3.18	1.25	4.0	^b^	0.28
*C. butyricum* CWBI 1009	0.09	−0.91	4.21	8.63	2.91	0.35
*C. pasteurianum* DSM525	4.96	−10.63	2.62	10.22	1.79	0.42
*C. felsineum* DSM 794	3.77	−28.62	8.98	1.04	^b^	0.24
Co-culture 1	1.06	−14.47	0.12	6.76	2.08	0.95
Co-culture 2	−0.42	−4.08	3.19	14.08	1.6	1.3
Co-culture 3	9.04	−6.49	8.22	17.81	2.32	1.05

^a^ Maximum biogas production rate.

^b^ This strain produced H_2_ mainly from the lactate and/or formate present in the medium, therefore it was not possible to correctly calculate the H_2_ yield per mole of hexose consumed.

*E. coli* and other Enterobacteriaceae can produce hydrogen from formate by the action of an enzymatic complex called formate:hydrogen lyase (FHL). However, none of the *Clostridium* strains studied has been shown to possess this type of enzyme. Surprisingly, another putative trimeric [FeFe] hydrogenase complex has been found in the genome of *C. beijerinckii* and it seems to be associated with a formate dehydrogenase-like protein [32]. This could explain the ability of *C. beijerinckii* to utilise formate for H_2_ production. However the enzyme activity has never been studied in this strain. A similar enzymatic complex was reported for *Eubacterium acidaminophilum,* and indeed the bacterium was shown to couple formate oxidation to H_2_ production [46].

In case of the co-cultures, the highest H_2_ yield of 2.32 mol H_2_/ mol hexose was obtained for co-culture (3) with *C. butyricum* and *C. pasteurianum.* Interestingly, the yield obtained for *C. butyricum* grown as a pure strain culture on starch was higher (2.91 mol H_2_/ mol hexose), but the biogas production rate tripled when the strain was combined with *C. pasteurianum* in a co-culture (Table 4). A comparison of the H_2_ yields obtained during glucose and starch sequences showed that, for starch, the H_2_ yields were higher for co-culture (1) with *C. pasteurianum* and *C. felsineum*, and co-culture (3) with *C. butyricum* and *C. pasteurianum*, probably due to the lower biogas production rates and the resulting lower H_2_ partial pressure, as was shown for the pure strains. Indeed, the biogas production rates during the starch batch fermentations ranged from 0.95 (co-culture 1) to 1.3 (co-culture 2) L biogas/ h, and except for the co-culture (2) they were 30% lower compared to the previous glucose batch fermentation.

Recently Quéméneur *et al*. showed, by performing a series of H_2_-producing batch fermentations, that both the carbohydrate chain length and alpha- or beta-linkage have an impact on H_2_ production [47]. In their work the H_2_ yields decreased from 1.84 (fructose fermentation) to 1.38 mol H_2_/ mol hexose (maltotriose fermentation) with the increasing chain length of the substrate. By contrast, the three co-cultures used in our study were characterized by equal or even higher H_2_ yields when grown on a polysaccharide (starch) compared to a monosaccharide (glucose). However different experimental conditions were applied in the two studies and the use of 500 ml glass bottles without pH control versus 20 L batch reactors operating at fixed pH may explain the differences in findings.

From the fermentation end-product analysis several observations can be made about the different strains’ and co-cultures’ metabolisms during the starch batch fermentation. A radical decrease in acetate production by a factor of 2.7 and 4.3 was found for pure strains of *C. butyricum* and *C. pasteurianum*, respectively (Table 4). Butyrate production was also shown to be lower for *C. beijerinckii* and *C. butyricum,* but it did not change significantly for *C. pasteurianum*. By contrast for the co-cultures fermenting starch a 1.3- and 2.1-fold increase in butyrate production was observed for co-cultures (2) with *C. butyricum* and *C. felsineum,* and (3) with *C. butyricum* and *C. pasteurianum*, compared to the previous batch sequence with glucose. At the same time the amount of acetate produced from starch decreased 2.5-fold and as much as 50-fold for co-cultures (2) with *C. butyricum* and *C. felsineum* and (1) with *C. pasteurianum* and *C. felsineum*, respectively. Interestingly the lactate produced during the former batch sequences with glucose was consumed in this step by the pure strains and by the three co-cultures. The highest rates of lactate consumption were observed for co-cultures (1) and (3), suggesting that *C. pasteurianum* may have been the main lactate consumer. Indeed this strain consumed the highest amounts of lactate during the starch fermentation stage when grown as a pure strain culture (Table 4).

Typical lactate consuming species, *e.g*. *Clostridium propionicum,* produces propionic and acetic acids together with CO_2_ during lactate fermentation, but no H_2_ is generated via this pathway [48]. However the lactic acid present in a starch containing medium, undergoes a different, not completely understood metabolic pathway, resulting in H_2_ production together with the formation of butyrate as the major metabolite [49]. Recently, Matsumoto and co-authors proposed a similar pathway, where one mol of acetate reacted with two moles of lactate giving in turn H_2_, CO_2_ and butyrate as the main products [33]. Indeed a radical decrease in molar acetate concentration was characteristic for the pure strains and the three co-cultures grown on starch, compared to the previous glucose fermentation stage (Table 4).

### Longer-term evaluation of H_2_ production using co-culture (3) with *C. butyricum* and *C. pasteurianum* and a glucose substrate in a SBR at fixed pH 5.3

As shown above, the *Clostridium* strains analysed in this study, when combined into two-strain artificial co-cultures, proved capable of producing H_2_ at significantly higher rates without negatively affecting the final H_2_ yield. Therefore, to further evaluate their H_2_ producing abilities, co-culture (3) was used as a model culture, since it had been shown to combine the best H_2_ yield and biogas production rate, during the glucose batch fermentation under controlled pH conditions. The co-culture was grown over a longer period of time (13 days), by extending the operating mode to 7 sequences. The fermentation was carried out at the fixed optimal pH value of 5.3, using glucose as a carbon source.

**Figure 4:**
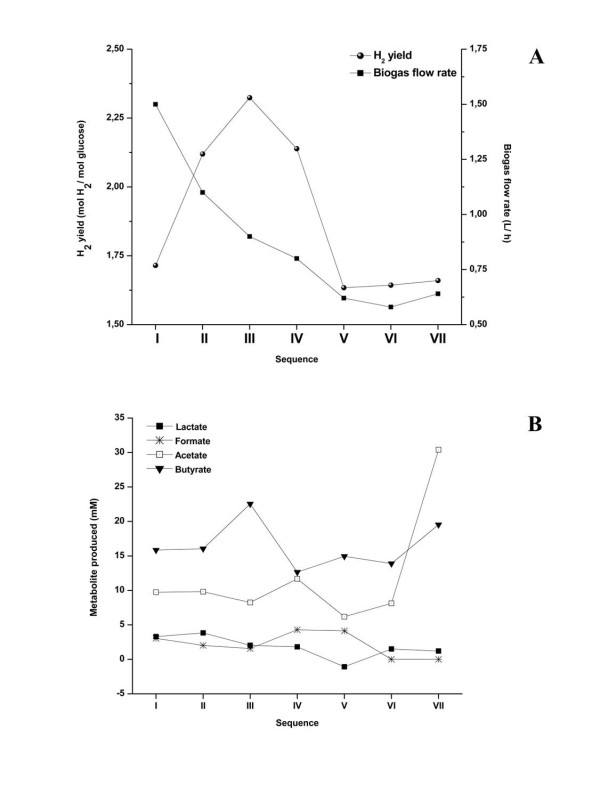
**Hydrogen yields and production rates (A) and fermentation end products (B) obtained for co-culture (3) with** ***C. butyricum*** **and** ***C. pasteurianum*** **during a 13-day glucose fermentation in a SBR.**

A series of experiments involved 7 sequences of removal/ addition of 12% (2 L) of the MDT medium, ensuring a glucose concentration of 5 g/ L after each removal/ addition. The sequences followed the initial batch culture and each sequence was started after the complete consumption of the previously added glucose. The biogas production rates and H_2_ yields were calculated for each sequence (Figure 4A), and the H_2_ content of the biogas was estimated to be between 62 and 65%. The biogas production rate measured during the first sequence was similar to that recorded during the batch glucose fermentation at fixed pH (1.45 L biogas/ h; Table 3). However, it was decreasing progressively and reached 0.8 L/ h at the end of sequence 4 (Figure 4A). During the last three sequences the biogas production rate stabilized at 0.6 L/ h. This decrease was probably due to the inhibitory effect of the accumulation of volatile fatty acids (VFAs, mainly butyrate and acetate), which was 5 ±0.46 fold higher at the end of the experiment than at the end of the first batch sequence. Acetate and butyrate represented 95.4% of the total VFAs present in the medium at the end of sequence 7, with minor amounts of lactate detected (4.6% of the VFAs). The production of the fermentative metabolites was similar to that for the previous batch glucose fermentation at controlled pH for the same co-culture (Figure 4B, Table 3). Formate was produced at low rates (3 mM ±1.09) and only during the first five sequences. Ethanol production was not detected. The distribution of the metabolites confirmed the relationship between high hydrogen yield and butyrate formation. The H_2_ yield increased from 1.71 to 2.32 mol H_2_/mol hexose during the first three sequences. It subsequently decreased after the fourth sequence and then stabilized at 1.63 mol H_2_/mol hexose during the last three sequences. Similarly, during the first three sequences the molar concentration of butyrate increased from 15.85 mM to 22.54 mM. When the hydrogen yield decreased after the fourth sequence the production of butyrate decreased to 12.64 mM. Similar correlation between the hydrogen yield and the production of butyrate had been previously observed [50,51]. However, such a correlation can only hold if the PFOR pathway dominates compared to the PFL pathway, generating higher amounts of the reduced Fd necessary for H_2_ production (Figure 2).

Although the maximal H_2_ yield obtained in this experiment represents less than 60% of the maximum theoretical yield that can be obtained by dark fermentation (4 mol H_2_/ mol glucose), only a few studies have reported higher yields, sometimes using much more sophisticated and expensive systems [39]. For example, employing a two-stage process combining thermophilic starch hydrolysis and dark fermentation, Chen *et al*. obtained a yield of 2.4 mol H_2_/mol hexose (reported as 13.2 mmol H_2_/g glucose) for *C. butyricum* CGS2 [52]. In another study a H_2_ yield of 2.79 mol H_2_/ mol sucrose was reported for an acclimated, mesophilic sewage sludge grown in an anaerobic SBR [53]. Interestingly, when combining *C. butyricum* in a co-culture with immobilized cells of *Rhodopseudomonas faecalis,* a H_2_ yield of 4.134 mol H_2_/ mol glucose was reported [54]. This indicates that the development of a two stage system, combining an artificial co-culture composed of *Clostridium spp.* with a photosynthetic H_2_-producing bacterium, could additionally increase the final H_2_ yield over the theoretical maximum of 4 mol H_2_/ mol glucose that can be reached by clostridia.

### Species dynamics for co-culture (3) with *C. butyricum* and *C. pasteurianum* monitored by qPCR during the 13 days of glucose fermentation in the SBR

A sequenced or a continuous operational mode for H_2_ production is necessary for its future industrial application [39]. However the behaviour and the genetic composition of a defined artificial co-culture can change over time, thus affecting the final H_2_ production.

**Figure 5:**
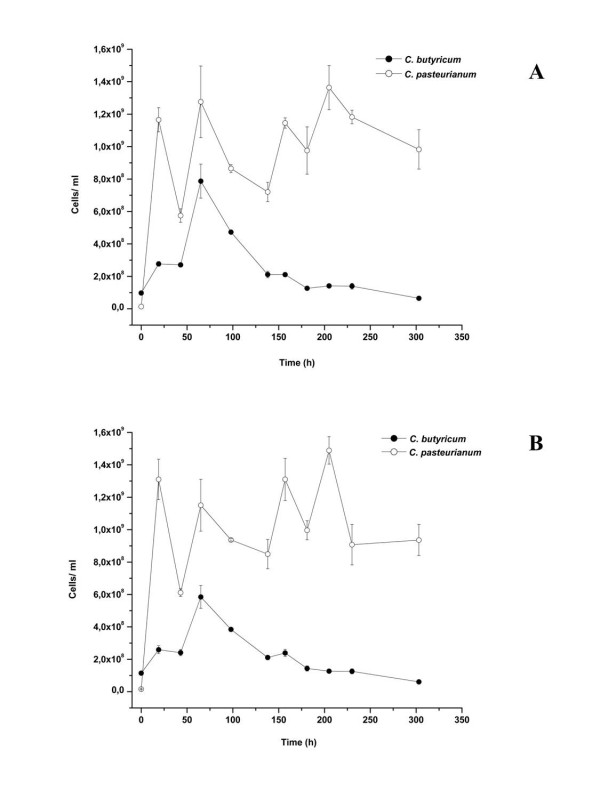
**Structural dynamics of co-culture (3) with*C. butyricum and C. pasteurianum*during a 13-day glucose fermentation in a SBR.** Cell numbers obtained from qPCR analysis based on *recA* (A) and *gyrA* (B) gene quantification. Sequence I (0–25 h), II (25–65 h), III (65–98 h), IV (98–138 h), V (138–181 h), VI (181–230 h), VII (230–303 h).

Therefore, in addition to the fermentation characteristics, the species dynamics of co-culture (3), with *C. butyricum* and *C. pasteurianum*, was studied during the 13-day glucose fermentation in the SBR. This monitoring was carried out using a previously optimized qPCR approach [17], and targeting the *recA* and *gyrA* genes from *C. butyricum* and *C. pasteurianum*. The qPCR assays, carried out with samples taken during each sequence step (at the beginning and/ or at the end of each sequence), revealed clear changes in the quantitative composition and the dominance of the community members. When performing the qPCR with both *recA* and *gyrA* primer sets the amplification results were similar and comparable to each other (Figure 5A and 5B). At the time of the inoculation of the bioreactor (sequence 1) *C. butyricum* constituted 87.6% ±0.06 of the co-culture population, whereas *C. pasteurianum* was much less abundant (12.39% ±0.06). However, *C. pasteurianum* had already become largely dominant after 19 h of incubation, accounting for 82% ±1.3 of the population, while *C. butyricum* had declined to 17.86% ±1.3. During the course of the experiment the number of *C. pasteurianum* cells present in the co-culture continued to increase. The highest proportion of *C. butyricum* was detected during the first four sequences when this species comprised 32.76% ±2.3 of the co-culture. Surprisingly, the highest hydrogen yield of 2.32 mol H_2_/mol glucose was also measured during the second sequence. The number of cells of *C. butyricum* started to decline slowly from the fifth sequence, representing only 6.13% ±0.05 of the total population after 13 days.

In general, as shown by qPCR, both organisms coexisted in the co-culture and no species was completely displaced by the other, despite the fact that there was only one carbon source. A similar situation was previously observed for the two closely related *Caldicellulospiruptor* species, combined in a H_2_ producing co-culture and grown under nutrient limiting conditions [13]. *C. saccharolyticus* which was in a minority during the batch start-up phase (less than 5% of the total population), overgrew *C. kristjanssonii* during the glucose and mixed sugar fermentations. Nevertheless none of the species was completely washed out from the co-culture. The occurrence of (complex) interspecies interactions between the microorganisms is hypothesized to be a key factor enhancing their stable co-existence in a population [13]. It has been suggested that under nutrient limitation a minority species can become metabolically active, and that this activity can be crucial for the survival of the whole population [10]. However further studies, including research on the species interactions and the metabolite trading between the bacterial members in a co-culture, are needed to better understand the behaviour of the different strains in a H_2_-producing artificial community.

## Conclusions

Artificial co-cultures composed of different *Clostridium* species were shown to offer better performance in terms of efficient H_2_ production than the corresponding pure strain cultures. In contrast to the pure strain cultures, of which only *C. butyricum* and *C. pasteurianum* were able to partially hydrolyse starch, the three co-cultures completely consumed the starch without any need for pre-treatment. Surprisingly, all the *Clostridium* strains and co-cultures studied were shown to efficiently reconsume the lactate present in a starch-containing medium. Additionally, *C. beijerinckii* appeared to produce H_2_ by re-oxidizing the previously produced formate. The utilization of lactate and formate for H_2_ production by different *Clostridium spp* suggests the existence of novel pathways leading to H_2_ in this genus.

The hydrogen yields obtained in this study by mesophilic fermentation of glucose and starch were amongst the highest reported to date for *Clostridium spp.,* cultured in a large lab-scale bioreactor (20 L). A comparison of the use of glucose or starch as a fermentation substrate resulted in contrasting data. Grown on starch the pure *Clostridium* cultures and the co-cultures provided higher yields compared to glucose fermentation. By contrast higher biogas production rates were reported when glucose was used as the carbon source.

Interestingly, co-culture (3), with *C. butyricum* and *C. pasteurianum,* produced H_2_ efficiently during 13 days of glucose fermentation in a SBR. Moreover both species co-existed stably in the co-culture during this time, despite the fact that there was only one carbon source available. Additionally, no species was completely displaced by the other, suggesting that positive interactions between the community members were at least as important as the simple competition for nutrients.

Future studies to improve substrate utilisation and metabolite trading should focus on the utilisation in H_2_ production of artificial co-cultures with increasing species complexity. In addition, the use of cellulose should be of prime interest since it would ensure the sustainability of the cheap carbon source. Therefore, the addition of a cellulose hydrolysing species, *e.g. Clostridium cellulolyticum,* to the co-culture should be considered.

## Methods

### Bacterial strains and culture conditions

The type strains of *C. beijerinckii* DSM 1820, *C. pasteurianum* DSM 525 and *C. felsineum* DSM 794 were purchased from DSMZ (Deutsche Sammlung von Mikroorganismen und Zellkulturen). *C. butyricum* CWBI 1009 was previously isolated from a hydrogen-producing sludge [21]. The strains were anaerobically maintained in MDT medium (5 g glucose, 5 g casein peptone, 0.5 g yeast extract, 1.2 g KH_2_PO_4_, 5.1 g Na_2_HPO_4_, 0.5 g MgSO_4_ x 7H_2_O and 0.5 g L-cysteine) as previously described [17]. Before the bioreactor inoculation the strains were individually sub-cultured for 24 h at 30°C under nitrogen atmosphere in 5 L glass bottles filled with MDT medium and equipped with silicone tubing and gas filters for sterile liquid transfer and outlet gas sterilisation. To initiate a co-culture an equal volume (L) of the two respective strain cultures was used.

### Bioreactor setup and sampling

All fermentations were carried out in MDT medium in a 20 L stainless steel tank bioreactor (Biolafite) equipped with a shaft with three Rushton impellers (four blades, diameter 100 mm, height 20 mm), 0.2 um gas filter, butyl septum and tubing for temperature regulation, gas inlet, gas outlet and medium removal/addition. After sterilization (120°C, 20 min) glucose or starch (rice starch, BENEO-Remy) were added sterilely and the medium was purged with nitrogen. The medium was continuously stirred at 100 rpm and the pH was maintained automatically at a given value using 3 N KOH (except for the unregulated pH fermentation). Finally 1 L of each (co-) culture was added to give a final working volume of 18 L. For the sequenced-batch mode after an initial growth in batch mode 2 L (3 L) of the medium were removed and the bioreactor was fed with 2 L (3 L) of fresh medium containing sufficient glucose to provide a final concentration of 5 g/ L in the bioreactor. Gas samples from the bioreactor headspace were regularly analysed. Culture samples were withdrawn to monitor the co-culture dynamics and analyse the metabolites. Cells were harvested by centrifugation at 13 000 x g for 1 min and the cell lysates were prepared as previously described [17]. The supernatant was further filtrated through a 0.2 μm cellulose acetate filter (Minisart Sartorius) and analysed for sugar consumption and metabolite formation.

### Experimental procedure

A three-step strategy was developed to characterize the hydrogen production of the pure strains and co-cultures with glucose and starch as alternative fermentative substrates. The first batch fermentation was carried out with unregulated pH and glucose at a final concentration of 5 g/ L. The initial pH was set at 7.3 and was allowed to decrease progressively as acidic metabolites accumulated. The optimal pH was defined as the value corresponding to the maximum biogas production rate. During the next sequence 3 L of the medium were replaced with the same volume of a fresh MDT medium containing glucose at a final concentration of 5 g/L. During this stage the pH was fixed at the optimal value by the automatic addition of sterile 1.5 N KOH. During the third sequence 3 L of the medium were replaced with the same volume of a fresh MDT medium containing starch at a final concentration of 5 g hexose/L. The pH was controlled during this stage. The determination of the optimal pH value by a multi-stage approach was performed as previously described [21].

### Analytical methods

The flow rate of the biogas produced in the bioreactor headspace was continuously measured with a wet flow meter (Ritter Gas meter Drum type TG01) connected to a computer running Rigamo data acquisition software (V1.30-K1). The proportion of hydrogen gas was determined using a gas chromatograph (GC) (Hewlett-Packard 5890 Series II) fitted with a thermal conductivity detector (TCD) and a 30 m x 0.32 mm GAS PRO GSC capillary column (Altech) in series with a 20 m × 0.25 mm CarboPLOT P7 column (Chrompak). The temperatures of the injection, TCD chambers and the oven were maintained at 90°C, 110°C and 55°C respectively. Nitrogen was used as the carrier gas in the column at a flow rate of 20 mL min-1. The HPLC analysis was carried out using an Agilent 1110 series (HP Chemstation software) as previously described [21].

### qPCR analysis

Sequences of species-specific primers targeting the *recA* and *gyrA* genes of *C. butyricum* and *C. pasteurianum* were as previously described (Table 5), [17]. PCR amplification was carried out with a Mini Opticon (BioRad). The DNA template was prepared as previously described [17]. For bacterial quantification 1 μl of washed cell suspended in TE^-4^ buffer was added directly to the PCR mix, as described previously [17]. The total volume of the PCR mix was 25 μl. The qPCR reactions specific for *recA, gyrA* consisted of 1 X PCR mix (ABsolute™ Blue QPCR SYBR® Green Fluorescein Mix, Thermo Scientific) and each primer (HPLC cleaned; Biomers, Germany) at a final concentration of 150 nM. The qPCR cycling conditions were as follows: initial denaturation at 95°C for 15 min followed by 40 cycles of denaturation at 95°C for 30 s, annealing at 60/57°C for 30 s and elongation at 72°C for 30 s. Each sample was analyzed in triplicate. No-template control was included in each run. The specificities of the amplifications were verified at the end of each qPCR reaction by performing the melting curve analysis. The standard curve was prepared as previously described [17]. The reaction efficiency was calculated as factor specific [55], using the equation: E = 10^-1/slope^ ( Additional file 1: Table S1).

**Table 5:** qPCR specific primers used in this study

**Strain**	**Primer name and sequence (5′ → 3′)**	**AT**^**a**^**(° C)**	**size (bp)**	**Accession number**
*C. butyricum* CWBI1009	**RecA-butF** AAGCATTAGTGCGTTCTGGAG	60	97	HQ433355
	**RecA-butR** GAATCTCCCATTTCCCCTTC			
	**GyrA-butF** AGCAATGGGTAGAACTGCATC	60	95	HQ433358
	**GyrA-butR** ATTCTTCGCCATCAACTGCT			
*C. pasteurianum*	**RecA-pastF** CTCATGTGGGACTTCAAGCA	60	150	HQ433356
DSM525	**RecA-pastR** CACCAGGTGTTGTTTCTGGA			
	**GyrA-pastF** AATGCATCTGGGGTAAGAGG	57	91	HQ433359
	**GyrA-pastR** CCACAAGTACATCCTTTTCAACA			

^a^ AT-PCR annealing temperature (° C).

## Abbreviations

Cq, Threshold value; E, qPCR reaction efficiency; Fd, Ferredoxin; GAPDH, Glyceraldehyde 3-phosphate dehydrogenase; gyrA, DNA gyrase; PFL, Pyruvate:formate lyase; PFOR, Pyruvate:ferredoxin oxidoreductase; qPCR, Quantitative real–time PCR; recA, DNA recombinase; SBR, Sequencing batch reactor; VFA, Volatile fatty acids.

## Competing interests

The authors declare that they have no competing interests.

## Authors’ contributions

JM performed the fermentation experiments, analyzed the results and helped to draft the manuscript. MC carried out the molecular analysis, analyzed the results and wrote the manuscript. CH and AW helped to draft the manuscript and participated in the coordination of the study. BJ, SH and PT participated in the coordination of the study. All the authors read and accepted the final version of the manuscript.

## Supplementary Material

Additional file 1 Details of the qPCR analysis. Click here for file
