# Supplementary material for: Fermentative hydrogen production from glucose and starch using pure strains and artificial co-cultures ofClostridium spp
Source: Biotechnol Biofuels. 2012 May 22;5:35. doi: 10.1186/1754-6834-5-35 (PMC3474151; doi:10.1186/1754-6834-5-35)
Supplement: Additional file 1 — Details of the qPCR analysis. [file 1754-6834-5-35-S1.pdf]

Supplementary Table 1

Details of the qPCR results obtained during quantification of the bacteria cell numbers in co-culture 3 with *C. butyricum* and *C. pasteurianum,* during glucose fermentation in a SBR.

	Reaction equation	r^2
C. butyricum CWBI 1009		
gyrA	y = -3.27x + 36.79	0.999
recA	y = -3.27x + 37.20	0.998
C. pasteurianum DSM 525		
gyrA	y = -3.232x + 38.33	0.999
recA	y = -3.328x + 38.69	0.999
